# Lumpy skin disease: history, current understanding and research gaps in the context of recent geographic expansion

**DOI:** 10.3389/fmicb.2023.1266759

**Published:** 2023-11-02

**Authors:** Ali Mazloum, Antoinette Van Schalkwyk, Shawn Babiuk, Estelle Venter, David B. Wallace, Alexander Sprygin

**Affiliations:** ^1^Federal Center for Animal Health, Vladimir, Russia; ^2^Agricultural Research Council – Onderstepoort Veterinary Institute, Onderstepoort, South Africa; ^3^Department of Biotechnology, University of the Western Cape, Bellville, South Africa; ^4^National Centre for Foreign Animal Disease, Canadian Food Inspection Agency, Winnipeg, MB, Canada; ^5^College of Public Health, Medical and Veterinary Sciences, Discipline Veterinary Science, James Cook University, Townsville, QLD, Australia; ^6^Department of Veterinary Tropical Diseases, Faculty of Veterinary Science, University of Pretoria, Onderstepoort, South Africa

**Keywords:** lumpy skin disease, transmission, EPI – epidemiology, phylogeny, recombination

## Abstract

Lumpy skin disease is recognized as a transboundary and emerging disease of cattle, buffaloes and other wild ruminants. Being initially restricted to Africa, and since 1989 the Middle East, the unprecedented recent spread across Eurasia demonstrates how underestimated and neglected this disease is. The initial identification of the causative agent of LSD as a poxvirus called LSD virus, was well as findings on LSDV transmission and epidemiology were pioneered at Onderstepoort, South Africa, from as early as the 1940s by researchers such as Weiss, Haig and Alexander. As more data emerges from an ever-increasing number of epidemiological studies, previously emphasized research gaps are being revisited and discussed. The currently available knowledge is in agreement with the previously described South African research experience that LSDV transmission can occur by multiple routes, including indirect contact, shared water sources and arthropods. The virus population is prone to molecular evolution, generating novel phylogenetically distinct variants resulting from a diverse range of selective pressures, including recombination between field and homologous vaccine strains in cell culture that produce virulent recombinants which pose diagnostic challenges. Host restriction is not limited to livestock, with certain wild ruminants being susceptible, with unknown consequences for the epidemiology of the disease.

## 1. Introduction

The previous decade has seen an unprecedented increase in scientific reports and publications on lumpy skin disease virus (LSDV), mainly due to its spread into new geographic regions and its adverse economic impacts on a global scale leading to greater interest in the disease and the causative agent (Bianchini et al., 2023). LSDV is a double-stranded DNA (dsDNA) poxvirus virus from the genus *Capripoxvirus*, transmitted in a similar manner to other capripoxviruses, i.e., sheeppox- (SPPV) and goatpox- (GTPV) viruses, *via* indirect contact and arthropod bites (Sprygin et al., 2019a; Nesterov et al., 2022; Shumilova et al., 2022a,b), although the most efficient route of transmission for SPPV and GTPV is direct and indirect contact whereas the main mode of transmission of LSDV is mechanical *via* blood-feeding arthropod vectors (Hamdi et al., 2021). It has warranted more focused attention since exceeding the previous limited geographic range in Africa, and then the Middle East, moving rapidly within the last decade into Turkey, Europe, Russia and Asia (Wainwright et al., 2013; Al-Salihi and Hassan, 2015; Ben-Gera et al., 2015; Ripani and Pacholek, 2015; Ince et al., 2016; Tasioudi et al., 2016; Zeynalova et al., 2016; Sameea Yousefi et al., 2017; Abutarbush and Tuppurainen, 2018; Mercier et al., 2018; Sprygin et al., 2018b; Manić et al., 2019). Furthermore, recombinant lineages, not sharing clonality with LSDV from the Middle East and Africa, have spread into China and many neighboring countries in eastern Asia, including Mongolia, Vietnam, Cambodia, Laos, Thailand, Malaysia, Singapore and Indonesia (Tran et al., 2021; Huang et al., 2022; Khoo et al., 2022; Sariya et al., 2022; Sprygin et al., 2022; Tsai et al., 2022; Krotova et al., 2022a).

Recent work already highlighted several knowledge gaps, including the development of cost-effective, safer and more effective vaccines that can Differentiate Infected from Vaccinated Animals DIVA, the efficacy of vaccines and the immunological protection of vaccines (including immunogenic epitopes for a protective response), direct and indirect transmission of capripoxviruses and the role of various arthropod vectors, and the development of improved diagnostic tests (including pen-side and CRISPR/Cas assays) (Tuppurainen et al., 2017; Sprygin et al., 2019c; Haegeman et al., 2020; Tuppurainen et al., 2021; Jiang et al., 2022; Liao et al., 2022).

This review will update and critically revisit the major gaps and limitations on transmission. In addition, this review describes a comprehensive historical description of observations on LSDV from South Africa (RSA) obtained from the Onderstepoort Veterinary Institute’s archives not previously accessible in electronic format.

## 2. Geographic distribution of LSDV

Lumpy skin disease (LSD) was first reported in 1929 in the Mazabuka, Lusaka, and Kufue districts of Northern Rhodesia (Zambia), where it was called pseudo-urticaria (MacDonald, 1931; Morris, 1931). In the absence of the known causative agent, the disease was predominantly characterized by firm circumscribed skin lesions, considered to be caused by insect stings or plant poisons, since transmission experiments using blood from infected animals constantly failed to produce disease symptoms (MacDonald, 1931; Le Roux, 1945). In 1943, an outbreak of the disease was reported in the Ngamiland district of Botswana (Von Backstrom, 1945), before its recognition in 1944 in the Groot Marico region of the Transvaal Province (North West Province) of South Africa. It spread rapidly across the country with outbreaks reported in all provinces, affecting up to eight million cattle. It was at this stage that the infectious nature of the disease was established, by inoculation of blood and tissue material from infected animals (Thomas, 1945). The disease then became endemic, with especially severe outbreaks being recorded in 1953–54, 1957 and 1962 (Thomas and Maré, 1945; Haig, 1957; Weiss, 1968). Southern Rhodesia (Zimbabwe) reported outbreaks in 1945 and by 1947 the disease had spread to Lesotho, Swaziland and Mozambique (Huston, 1945; Diesel, 1949; De Sousa Dias and Serra, 1956). By 1956, it was reported in Madagascar, Tanzania and Belgian Congo, whilst in December 1957, it was reported in the Nakuru district of Kenya (Haig, 1957; MacOwen, 1959). The LSDV strain isolated from Kenya in 1959 (NI-2490) was the first to be subjected to complete genome sequencing (AF325528) (Capstick and Coackley, 1961; Tulman et al., 2001). Concurrently, in 1957 in South Africa, Alexander et al. (1957) demonstrated the true causative agent of LSD, with the identification of a poxvirus. The Neethling strain of this virus, now called lumpy skin disease virus (LSDV), isolated from the Western Cape Province of South Africa, became the type strain. The complete genome of this isolate (Neethling/1957: OM793608), as well as four additional isolates from South Africa in the 1950s (Haden/1954: MW656252; Potter/1958: OM793606; Hoffmeyer/1958: OM793605; Fourie/1959: OM793607) were only recently sequenced and published (van Schalkwyk et al., 2022). Although viruses, NI-2490/1958 (Kenya) and Neethling/1957 (RSA), were circulating in their respective countries during the same period, they each belong to a different genetic cluster of LSDVs, based on recent sequencing evidence (to be discussed later in this review).

In the latter half of the 20th century, the disease became endemic to the majority of sub-Saharan African countries. In 1988, Egypt reported its first cases of LSD, followed by Israel in 1989. LSD then became endemic in North Africa and the Middle East, resulting in its subsequent spread across Turkey (2013) to the European Union (2015), the Balkans and Russia (2015). Complete genome sequencing was performed on various LSDV isolates from Israel (155,920/Israel/2015; KX894508), Turkey (Pendik/Turkey/2014; MN995838), Greece (Evros/Greece/2015; KY829023), Russia (Dagestan/2015; MH893760), Serbia (Bujanovac/Serbia/2016; KY702007) and Bulgaria (210-249/Bulgaria/2016; MT643825), obtained during outbreaks in these countries (Mathijs et al., 2016; Toplak et al., 2017; Agianniotaki et al., 2017a,b; Sprygin et al., 2019b). Sequence comparisons between these LSDVs indicated a high percentage of sequence identity amongst them, pointing toward a shared origin of the LSDV isolates involved in these outbreaks (van Schalkwyk et al., 2022).

The first novel recombinant strain was identified in Saratov, Russia, in 2017 (Sprygin et al., 2018b). This was subsequently followed by the identification and characterization of four additional unique, novel recombinants. These were isolated from non-typical outbreaks in Udmurtya, Russia, 2018 (Sprygin et al., 2020), Tuymen, Russia, in 2018 (Krotova et al., 2022b) and Xinjiang, China in 2019 (Ma et al., 2022). Recombinant viruses clustering with the LSDV identified in China have spread across China, Mongolia (2021), Vietnam (2020), Thailand (2021) and Eastern Russia (2022) (WAHIS, 2023). In 2019, the first outbreaks of LSD were reported in Bangladesh and India, but in contrast to the novel recombinants described in southeast Asia, the isolate responsible for these outbreaks was caused by a KSGPO-like vaccine strain (Roche et al., 2020; Sudhakar et al., 2020; Kumar et al., 2021). This parental strain of the KSGPO-like vaccine then spread from Bangladesh and India to Nepal, Myanmar, Sri Lanka, Pakistan and Afghanistan (Hasib et al., 2021; Pandey et al., 2021; Maw et al., 2022).

## 3. Host tropism of LSDV

Capripoxviruses have a narrow host tropism and are non-zoonotic viruses. SPPV and GTPV have a host tropism for sheep or goats, respectively, however there are some strains that can infect both sheep and goats. Furthermore, there is evidence that GTPV can infect closely-related wildlife with outbreaks in wild Red Serow (Dutta et al., 2019) and Himalayan goral (Bora et al., 2021). Although inoculation of some SPPVs in cattle can cause clinical signs of disease (Abutarbush and Tuppurainen, 2018), there have been no reports of SPPV or GTPV infecting cattle naturally in the field. This is best illustrated in countries endemic with SPPV or GTPV and free of LSDV, where cattle do not demonstrate a capripoxvirus-like disease. The primary host for LSDV is cattle with other hosts including the Asian water buffalo. LSDV does not cause clinical signs of disease in sheep or goats, however, some LSDVs can replicate in sheep and goats following inoculation (Zewdie et al., 2021). This is illustrated in regions such as southern Africa, which are endemic for LSDV and free of SPPV or GTPV, where sheep and goats do not show clinical signs of capripoxvirus disease. The one documented occurrence of LSDV in sheep in Kenya was most likely a rare event and surprising, as the clinical disease was mild with the absence of skin lesions (Davies, 1976). Furthermore, it is possible that the KSGPO-240 virus subsequently isolated was either contaminated or mislabeled during processing or storage in the laboratory, which went undetected since no molecular tests or genomic sequencing were available at the time. The KSGPO-240 virus used as a SPPV and GTPV vaccine, or inappropriately as a LSDV vaccine, is indeed a LSDV (Vandenbussche et al., 2016). Further surveillance of sheep and goats using virus isolation from swabs and tissues where LSDV is present is required to determine if sheep and goats can get infected with LSDV subclinically in field conditions.

In contract, LSDV has a broader host tropism, as illustrated by the isolation of LSDV genomic DNA from skin lesions from springbok, Oryx and giraffe, as well as the severe clinical reactions resulting from experimental infection of impala and giraffe (Young et al., 1970; Gelaye and Lamien, 2019; Dao et al., 2022). A nasal swab from an oryx was positive for LSDV by molecular methods (Molini et al., 2021), as well as suspected LSDV infection in a captive-bred Arabian oryx (*Oryx leucoryx*) from Saudi Arabia, although the latter was only serologically confirmed as a capripoxvirus (Greth et al., 1992). With the recent geographic expansion of the disease, clinical symptoms and molecular identification of LSDV have been identified in camels and free-tanging Indian gazelles (*Gazella bennettii*) in India in 2022 (Kumar et al., 2023; Sudhakar et al., 2023). Additionally, LSD caused high mortality in yaks in China (Li et al., 2023) and clinical signs in gaurs (*Bos gaurus*), Mainland serow (*Capricornis sumtraensis*) and banteng (*Bos javanicus*) in Thailand in 2021 (World Organization for Animal Health, LSD, FAQ, 2022). Given the history of LSD emerging in Africa, the major question arises as to which species is the natural host, from which the virus then jumped to cattle. To address the research gap in understanding the host tropism for capripoxviruses, a concerted effort using comprehensive surveillance is required to identify potential hosts. In support of this question, antibody surveillance of game animals in the Kruger National Park in South Africa was performed and in addition to the previously mentioned antelope species, antibodies against capripoxviruses were detected in African buffalo (*Syncerus caffer*) (Fagbo et al., 2014).

The majority of disease cases are reported in cattle. It has been observed that the breed, age, color and sex of the animal plays a role in the likelihood of the animal to be susceptible to the disease (Weiss, 1968; Bianchini et al., 2023). Despite these intrinsic factors, the disease could be controlled by restricting the movement of diseased animals and prevented through annual vaccination campaigns (Hunter and Wallace, 2001).

## 4. Transmission of LSDV

Since the first outbreaks of LSD in southern Africa, the predominant mode and mechanism of transmission has been a contentious issue. This is in part due to the nature of the disease, as well as different conditions under which it was described. The original outbreaks in South Africa in the 1940s were vastly different from what was observed in Kenya in the 1950s (Thomas and Maré, 1945; MacOwen, 1959). The South African outbreak spread across the country within a year, whilst in 12 months in Kenya it was confined to 58 farms in a 25 miles radius. This could be due to social political conditions, the individual countries’ preparedness and responses toward the disease or the difference in cattle populations, farming practices or viral strains (Henning, 1956; MacOwen, 1959). In contrast to the strict quarantine measures implemented by Kenya, the disease outbreaks in South Africa were congregated along the railways and roads, which fit into movement of animals as a source for new infections (Haig, 1957). The initial outbreak in Kenya could not be linked to the introduction of cattle, but the first farm did report the importation of fat-tailed sheep (MacOwen, 1959). Cases in Kenya were less severe compared to the ones described by Thomas and Maré (1945) in South Africa. The latter was observed to persist throughout the year with the highest incidence reported during the summer months. Although it abated during the winter, new outbreaks were reported from farms where heavy frosts were occurring, suggesting a possible alternative to vector-borne transmission (Haig, 1957; Weiss, 1968). Yet, since the control measures could not effectively prevent the spread of LSD and the disease was mostly prevalent during wet summer months, especially in low-lying areas, led researchers to surmise that insects play an important role in transmission (Von Backstrom, 1945; Diesel, 1949; Henning, 1956).

At the same time, Haig (1957) reported infection in the absence of insects, which is feasible, either directly or indirectly through fomites, where infected saliva could be involved. A number of outbreaks occurred many miles away from the known source of infection, which could be explained by cattle movement rather than insects (Haig, 1957). Additional evidence for alternative modes of transmission is provided by the ineffectiveness of insecticides in preventing the spread of LSD (Haig, 1957).

Different routes of transmission of LSDV have been reported under laboratory (experimental) conditions. The shedding of the virus in skin nodules of affected animals, infected saliva, ocular and nasal discharges and semen were reported as possible sources of virus. Also, transmission was achieved when naïve cattle were allowed to share a drinking trough with severely infected animals in insect-free facilities (Weiss, 1968; Ali et al., 2012). The prolonged excretion of LSDV in bovine semen, even in asymptomatic bulls, raises concerns for venereal spread (Irons et al., 2005) and transmission by artificial insemination of spiked semen has been established experimentally (Annandale et al., 2014), while intrauterine transmission was reported in the field (Rouby and Aboulsoud, 2016).

### 4.1. Vector transmission of LSDV

The inefficient transmission of LSDV in the absence of arthropod vectors has been reported (Carn and Kitching, 1995), which is indirectly supported by the seasonality of outbreaks being mainly linked to warm and rainy months, with much rarer occurrences in winter (Byadovskaya et al., 2022). Mechanical transmission by arthropods (some tick species, *Stomoxys*, *Aedes,* and *Culex*), has been assumed to be the most significant mode of viral transmission, with no evidence of active virus replication in insects or ticks (Tuppurainen et al., 2013a; Sprygin et al., 2019a,b,c). Supporting evidence for arthropod transmission includes the appearance of the disease several hundred kilometers away from the initial outbreak within a short time period, linked to the observation that the disease outbreaks are predominantly occurring during the rainy seasons and along water courses associated with an increase in insect activity. In addition, LSDV has spread into new regions at a significantly higher pace compared to SPPV and GTPV. These differences could be attributed to the ease of spread of LSDV by multiple vectors in contrast to SPPV and GPPV which are not transmitted efficiently by vectors over long distances but there is scope for that. Alternatively, the greater spread of LSDV into new regions might be related to movement of animals (especially where this is human-assisted), animal feed and/or products, which is in other words, fomite transmission. Weather changes, including cold spells and freezing winter conditions, adversely affect the insect vector populations and thus a reduction in LSDV transmission is observed, although outbreaks during the dry season and winter months have been reported in Africa (Haig, 1957; Davies, 1982).

Although live LSDV has been isolated as early as the 1960s from stable flies *Stomoxys calcitrans* and *Biomyia fasciata (Musca confiscata)* after feeding on infected cattle (Du Toit, 1965), vector transmission studies only started in the 1990s and are difficult to perform, with inconsistent results. In a more recent study, the intrathoracic injection of three *Stomoxys* species (*Stomoxys calcitrans*, *Stomoxys sitiens,* and *Stomoxys indica*) with LSDV was able to demonstrate the viability of the virus in the haemolymph (Issimov et al., 2020).

Female *Aedes aegypti* mosquitoes and *Stomoxys calcitans* flies have been demonstrated to have the ability to successfully transmit LSDV (Chihota et al., 2001; Issimov et al., 2020). In other studies, however, LSDV transmission from infected to susceptible cattle using mosquitoes (*Anopheles stephensi*), stable flies (*Stomoxys calcitrans*) and biting midges (*Culicoides nubeculosus*) was not achieved (Chihota et al., 2003).

The variations in experimental results are likely due to low levels of viraemia in the blood of infected animals contributing to the inefficient transmission of LSDV by biting flies feeding on blood, alone. It was suggested that biting flies have to feed on skin lesions, or nasal and ocular discharge, to obtain sufficient virus load for subsequent transmission to occur. Insects feeding on a subclinical animal are 97% less likely to acquire LSDV compared to feeding on an animal displaying clinical symptoms (Sanz-Bernardo et al., 2021).

The virus persists in the skin and could be isolated 38 days post-infection (Weiss, 1968), whilst viral DNA was detected using PCR in skin lesions for more than 90 days (Tuppurainen et al., 2005). The questions pertaining to the most suitable source and form of LSDV for obtaining vector-mediated transmission, i.e., the source of virus from either blood and/or skin, and intracellular mature virus (IMV) and/or the extracellular enveloped virus (EEV) forms, still remain unanswered. There is little doubt that cattle with skin lesions allow for transmission of LSDV *via* vectors. As part of the successful control measures to contain LSD spread in Europe, cattle with skin lesions were stamped out, whilst in 1958 strict quarantine measures inhibited spread of LSD in Kenya to 25 square-miles, without the control of insect populations (MacOwen, 1959). However, the question concerning the capabilities of viremic cattle without skin lesions to transmit LSDV in the field remains unanswered and additional laboratory and field experiments are required to investigate the transmission of LSDV from asymptomatic infected animals to uninfected animals. Recently, it was demonstrated that LSDV genomes could be detected using real-time PCR in ear notch and skin biopsies of subclinically-infected cattle, further complicating the issue (Van Campe et al., 2021). Until in a recent study performed by Haegeman et al. (2023a,b), the transmission of LSDV from sub-clinically infected animals by *Stomoxys calcitrans* was demonstrated. Authors of the study performed an *in vivo* transmission study with 13 donors, experimentally inoculated with LSDV, and 13 naïve acceptor bulls whereby *S. calcitrans* flies were fed on either subclinical- or preclinical-infected donor animals. Transmission of LSDV from subclinical donors showing proof of productive virus replication but without formation of skin nodules was demonstrated in two out of five acceptor animals, while no transmission was seen from preclinical donors that developed nodules after *S. calcitrans* flies had fed. Interestingly, one of the acceptor animals which became infected developed a subclinical form of the disease (Haegeman et al., 2023a,b).

LSDV DNA has been detected in *Culex quinquefasciatus, Anopheles stephensi* and *Culicoides nubeculosus* species after feeding on infected cattle or an infectious blood meal (Chihota et al., 2003), as well as in field-caught pools of *Culicoides punctatus* (Sevik and Dogan, 2017). However, transmission studies of the virus to susceptible animals have not been confirmed for these insects. In a more recent experimental study, *Aedes aegypti*, *Culex quinquefasciatus*, *Stomoxys calcitrans,* and *Culicoides nubeculosus* acquired the virus and could retain virus for more than 8 days (Sanz-Bernardo et al., 2021), although it was not possible to demonstrate viral replication in these vectors and the authors concluded that *S. calcitrans, C. nubeculosus,* and *A. aegypti* can potentially be efficient mechanical transmitters of the virus.

The life cycle of many species of ticks (such as *Amblyomma* spp.) enables them to be competent disease vectors. Ticks may feed on several different mammal, bird and reptile species. Those feeding on birds or mammals may be effective transmitters of a virus such as LSDV in the field. The life cycles of ticks long and individual life cycle stages may survive for years without a blood meal (Tuppurainen et al., 2011). LSDV was detected in different life cycle stages of various tick species and can survive in tick populations throughout the entire life cycle of the tick. The viral DNA presence also persists in tick tissues that do not undergo histolysis (synganglia and hemocytes) and in tissues that emerge anew during the molting such as reproductive organs (Tuppurainen et al., 2011, 2013b; Lubinga et al., 2014a,b). This may explain the apparent overwintering of the virus and the sudden reappearance of the disease after an absence of several years in tropical climate. However, experiments were carried out on African tick species and further studies are required to look at the vector-competency of tick species present in geographic regions of the Northern Hemisphere.

The maximum distance that LSDV can transmit by vectors is currently unknown. It is known that LSDV has spread over waterways in Southeast Asia and will likely continue to spread throughout the islands in the region (Das et al., 2021). Monsoons and cyclones in the region can likely increase the distance vectors can be spread. The risk of the virus entering Australia, a LSDV-free country, is high and natural transmission *via* insects, driven by wind, is currently predicted as the most likely mechanism by which it may enter. Bluetongue virus, a *Culicoides-*transmitted virus, has previously entered Australia from Southeast Asia *via* wind-borne infected *Culicoides* spp. (Boyle et al., 2012).

### 4.2. Non-vector borne and indirect transmission

The unprecedented spread of LSDV in Russia, in a northern and eastward direction, has exposed the existing gaps in the understanding of LSDV transmission including overwintering mechanisms in northern latitudes such as environmental contamination of grazing fields as can occur with SPPV and GTPV, spread outside of putative insect vector abundances, role of fomites, as well as differences in the distribution of LSD outbreaks between dairy farms compared to beef cattle farms (Byadovskaya et al., 2022; Singhla et al., 2022). The currently available epidemiological evidence suggests transmission by non-vector routes (Sprygin et al., 2020; Singhla et al., 2022; Shumilova et al., 2022a,b).

Kononov et al. (2020) demonstrated indirect transmission with the novel recombinant LSDV field strain, Saratov/2017, in line with previous observations of indirect transmission from South Africa, but in which a Neethling-type strain of virus was used. The genome of the recombinant Saratov/2017 is represented by a Neethling LAV strain as the major parent and Kenyan vaccine strain KSGP as the minor parent (Sprygin et al., 2018a). Recombination occurring in viruses has a tremendous influence on viral divergence, change of virulence and host-switching/expansion (Esposito et al., 2006; Smithson et al., 2014; Zhao et al., 2015; Sprygin et al., 2022), and thus further characterization of the novel Saratov/2017 strain was performed. Under controlled conditions in an insect-proof facility, the Saratov/2017 strain demonstrated an ability to transmit to in-contact animals sharing water and feed troughs. The in-contact animals became viraemic around 3 weeks after the onset of viraemia in inoculated animals. This was the first experimental demonstration that LSDV can transmit to other animals in an indirect manner. Importantly, sharing of water troughs was first raised as a risk factor in the early days of LSDV studies (Weiss, 1968), but this mode was overlooked due to its claimed inefficiency and was not pursued further (Sprygin et al., 2019a). Classical field LSDV strains (belonging to either cluster 1.1 or 1.2) are primarily spread by vectors feeding on cattles.

Following this observation, the Saratov/2017 and Dagestan/2015 strains were evaluated for transmission *via* oral feeding. The results obtained demonstrate Saratov/2017’s unique characteristics in relation to its ability to infect cattle following oral inoculation, while cattle inoculated orally with the non-recombinant Dagestan/2015 strain did not become infected. The direct comparison used in the study points to a clear difference in transmission efficiency between classical and recombinant isolates. Further studies are required to determine the impact of additional transmission mechanisms of LSDV in the field.

The LSD outbreak reported in 2019 in Russia during freezing winter conditions with snow, demonstrates the importance of transmission in the absence of vectors (Sprygin et al., 2020). Following genomic characterization, the recombinant strain isolated from the outbreak, designated Udmurtiya/2019, was identified as another novel mosaic strain in which the viral parental contributions were predominantly those of a KSGP vaccine strain, with a Neethling vaccine strain as a minor parent. Its transmission properties were evaluated and compared to Saratov/2017. It was observed that Udmurtiya/2019 can infect in-contact bulls in an indirect manner in a similar fashion to Saratov/2017 (Nesterov et al., 2022).

The reviewed literature demonstrates an intensified focus on understanding LSDV transmission. Classical field isolates belonging to Cluster 1.2 are actively examined in terms of putative vectors, while recombinant LSDV strains are being assessed for alternative modes of transmission (Chihota et al., 2001; Lubinga et al., 2014a; Wang et al., 2022). Recombinant LSDVs do exhibit improved oronasal transmission compared to classical field isolates, due to their altered genomes. Of paramount importance, two major pools of LSDV lineages must be treated separately: the classical lineage (cluster 1.2), restricted to Africa, Middle East and the recombinant lineages prevailing in Russia and South Eastern Asian countries (Krotova et al., 2022c). It would be counter-productive to generalize the global epidemiology as a single pool and, more importantly, to extrapolate conclusions drawn on classical field isolates onto recombinant lineages displaying unique features of indirect transmission similar to the LSD *capripoxvirus* counterparts, SPPV and GTPV.

The global virus population of LSDV is represented by multiple clusters and lineages (Krotova et al., 2022a). Prior to 2000, it is suspected that South African researchers worked with Cluster 1.1 isolates, whilst the recent findings involving arthropods were derived exclusively from Cluster 1.2 isolates (Paslaru et al., 2021; Sanz-Bernardo et al., 2021; van Schalkwyk et al., 2022). In contrast, recent experiments into indirect transmission mechanisms are solely based on recombinant vaccine-like isolates that occurred since 2017 in Russia, Kazakhstan, China and South Eastern Asian countries (Nesterov et al., 2022). Objectively considering the available findings, LSDV is contagious and vector-borne disease in agreement with past work in Onderstepoort institute that first delineated the nature of LSDV transmission. Moreover, being a capripoxvirus, LSDV can use the modes of transmission exhibited by sheep pox virus and goat pox virus, and poxviruses in general (Sprygin et al., 2018a; Nesterov et al., 2022). Further studies to determine the impact of both mechanical and oronasal transmission on transmission in the field are required.

### 4.3. Direct contact and migration of domestic or wild ruminants

Based on the recent findings using molecular epidemiology, it is evident that human-associated movement of animals plays the biggest role in the spread of the disease. This was initially observed by Diesel in 1949, indicating that LSD predominantly spread along the railways and motorways. Long distant and transboundary spread of the disease is due to the movement of infected animals, whilst short distance spread could be due to direct contact or mechanically *via* insects (Sprygin et al., 2019b). The impact of wildlife in the spread of the disease has not been investigated in-depth (Molini et al., 2021).

## 5. Control through vaccination

The uncontrollable spread of LSDV into new regions even with the use of vaccines demonstrates the need to understand how to implement a proper control plan. Unlike SPPV and GTPV which can be eradicated through stamping out, stamping out in the absence of vaccination has not been effective to eradicate LSDV. Europe first demonstrated that LSDV could be eradicated using vaccination in addition to other control measures (Calistri et al., 2020).

Several different live attenuated vaccines have been developed and used in cattle to protect against LSDV. These vaccines have been referred to as homologous when the vaccine derived from the same capripoxvirus (LSDV) and heterologous vaccines derived from a different capripoxvirus (SPPV or GTPV). It should be noted that there are differences in efficacy and safety between homologous vaccines as well as between heterologous vaccines with respect to efficacy, so care must be taken when describing the vaccine used (Haegeman et al., 2021a,b).

In South Africa, the Neethling/1957 isolate was serially passaged 61 times on lamb kidney cell monolayers and an additional 20 times in the chorioallantoic membranes of embryonated hen’s eggs to obtain an attenuated vaccine phenotype (Neethling-LW-1959) (van Rooyen et al., 1959). The complete genome of this live attenuated vaccine (LAV) has been determined (AF409138) and forms the basis for three commercial vaccines currently used in South Africa (Onderstepoort Biological Products [OBP], KX764645; Herbivac, KX764644 and MK441838; and, SIS-Lumpyvax, KX64643) (Kara et al., 2003; Mathijs et al., 2016; Lojkić et al., 2018; Douglass et al., 2019). In contrast, Kenya employed a heterologous vaccine strategy and combined viruses isolated from sheep and goats following serial passage attenuation steps (Davies et al., 1971) to develop a vaccine. The use of heterologous vaccines to protect against LSD are possible due to the cross-protection afforded by the three capripoxviruses, SPPV, GTPV and LSDV, considered as host-adapted genotypes of a single virus considering how names were originally given to pathogens affecting each host. A Kenyan vaccine known as Kenyan sheep-and-goatpox (KSGP) ovine-240 (KSGPO-240, later known as KS-1), were developed using limited passage in cell culture from field strains isolated from sheep and goats (Capstick and Coackley, 1961; as referred to by Davies, 1976; Davies and Otema, 1978; Davies and Mbugwa, 1985). Earlier work, using restriction fragment length polymorphisms of viral DNA and later confirmed using gene sequencing and complete genome sequencing, showed that the commercial vaccine based on KS-1 was actually LSDV, rather than SPPV or GTPV (KSGPO-240; KX683219) (Tuppurainen et al., 2014; Vandenbussche et al., 2016). These vaccines, as well as other SPPV and GTPV vaccines used to control outbreaks of LSD, were recently reviewed by Tuppurainen et al. (2021).

The Neethling-based live attenuated vaccines (LAV) developed in South Africa (Onderstepoort Biological Products), (Herbivac) and (SIS-Lumpyvax), have been used extensively in cattle since the development in the late 1950s of the Neethling-LW1959 vaccine. These vaccines can cause some side effects, i.e., skin reactions, following administration in cattle. Genetically, seven single nucleotide polymorphisms (SNPs) have been identified between the original Neethling (WC-RSA-1957) isolate and the Neethling LAV(LW1959) strain (van Schalkwyk et al., 2022). Currently, the genetic link between virulent and attenuated phenotype has not yet been established (van Schalkwyk et al., 2022).

A comparative study of LSDV Vaccines (Onderstepoort Biological Products OBP; South-Africa), Lumpyvax (MSD-Animal Health; South-Africa), Kenyavac (Jordan Bioindustries Center Jovac; Jordan), Herbivac LS (Deltamune; South-Africa) and Vaccin LSD Neethling O vivant (MCI Santé Animale; Morocco) was performed in cattle demonstrating the efficacy of these vaccines (Haegeman et al., 2021b).

The Kenyan sheep- and goatpox (KSGP) vaccines, which have been genetically characterized as LSDVs, have been used for vaccines against SPPV and GPPV (Vandenbussche et al., 2016). The KSGP vaccines are not completely attenuated and can cause clinical disease in cattle especially in dairy cattle (Yeruham et al., 1994). Furthermore the LSDV outbreaks in the Indian subcontinent from this virus (Sudhakar et al., 2022) demonstrate that this is a virulent LSDV which can cause outbreaks in naive cattle.

Comparing the genome sequence of the KSGP vaccine to the Neethling-based vaccines revealed disruption of kelch-like proteins 19 and 144 as well as 134 (B22R) in Neethling-LW1959 vaccines and only disruption of 134 (B22R) in the KSGP vaccine indicating molecular differences in attenuation (Biswas et al., 2019).

Eradication in Europe was achieved using LAV homologous vaccines from South Africa in addition to other control measures including stamping out of cattle with clinical disease (Calistri et al., 2020). Despite the same vaccines used in South Africa, LSDV remains endemic in the region. The reason for this is not the lack of vaccine efficacy but rather vaccine coverage that is not high enough to break the transmission cycle (Hunter and Wallace, 2001). Other factors which can influence the success of a vaccination campaign are ‘Vaccine breakdown’ or disease outbreaks have been linked to: Vaccination of animals that were already incubating the disease. Confusion of the disease with ‘pseudo lumpy skin’ disease (Allerton virus, BHV-2). LSDV infection in unvaccinated calves, after the disappearance of maternal antibodies after 3 months (Hunter and Wallace, 2001; Agianniotaki et al., 2018). Poor maintenance of the cold chain resulting in decreased vaccine efficacy. In addition, incorrect vaccination schedules – in winter - long before the ‘season’ virus/disease is not seen regularly lead to producers believing vaccination is not worth the effort. Furthermore, there are cultural perceptions where not all farmers believe in vaccination (Masemola et al., 2021). The infrequent or improper use of the vaccine – re-use of needles as well as the availability of some LSDV vaccines in the country, lead to inadequate vaccination coverage. In addition, the increased susceptibility of European high producing cattle breeds to LSDV compared to African cattle breeds may influence the decisions to have cattle vaccinated (Davies, 1991). All these factors demonstrate that eradication of LSDV requires a national mandate to be achievable.

Despite the value of vaccination in controlling and preventing the spread of LSDV, contaminated vaccines played a disastrous role in the spread of LSD. In 1945, isolation attempts of LSDV samples submitted to the ARC-Onderstepoort, resulted in the spread of the disease to stables where cattle designated for the maintenance and production of *Anaplasma centrale* were kept. Of the 83 cattle involved in the *A. centrale* trial 43 manifested with clinical symptoms of LSD, including two oxen bled for *A. centrale* blood vaccine days before the onset of clinical symptoms. The *A. centrale* blood vaccine was unfortunately already administered and 12 days later the vaccinated cattle displayed signs of LSD, indicating the first transmission of the disease through inoculation with infected blood samples (Henning, 1949).

The European Union utilized the Neethling-based LSD vaccines, commercially available from manufacturers in South Africa, to control the spread and subsequently eradicate the disease in the Balkans (Calistri et al., 2020). In contrast, Russia employed a heterologous vaccination strategy based on the NISKHI SPPV vaccine. Despite vaccination with attenuated Neethling/vaccine being prohibited in Russia, the latter was identified in outbreaks in the Orenburg, Bashkortostan and Samara regions in 2017 (Kononov et al., 2019). Kazakhstan employed a vaccination campaign between 2017 and 2019, using the commercial vaccine Lumpivax from KEVEVAPI (Nairobi, Kenya) and AU-PANVAC (Debre Zeit, Ethiopia) (Vandenbussche et al., 2022). Recently, this vaccine was reported to contain both Neethling/LW1959, KSGPO-like, as well as GTPV genetic material (Vandenbussche et al., 2022).

Recent examples of the spread of LSD through contaminated vaccines include the numerous novel recombinant strains isolated in Russia, Kazakhstan and China, linked to the contaminated Lumpivax vaccines administered in Kazakhstan (Vandenbussche et al., 2022). The vaccine contains the genetic material of both the Neethling-LW1959 and KSGPO-like vaccines, and was administered between 2017 and 2019. This coincided with the detection of five novel recombinant strains, each sharing various patterns of the parental strains, Neethling-LW1959 and KSGPO-like vaccines.

The first report of LSD in India was in 2019, with the detection of KSGPO-like strains circulating in the sub-continent. This vaccine virus spread from Bangladesh or India to Nepal, Sri Lanka and Pakistan, yet the introduction of this vaccine strain to the region is not yet known (Hasib et al., 2021; Pandey et al., 2021; Maw et al., 2022). It is possible that LSDV entered Bangladesh or India either through vectors, which is unlikely since there was no LSDV outbreak near the Indian region where the first outbreak of LSDV occurred, and the virus was also of African origin. Live LSD-infected cattle imported legally or illegally could also be a source of the virus. Since SPPV and GTPV are present in these countries, there is a market for vaccines for SPPV and GTPV and the potential for the illegal importation of vaccines. Thus, another possibility is the illegal importation of a KSGP vaccine and use in animals. It is speculated that this commercial vaccine against SPPV and GTPV was either administered to the corresponding animal species and subsequent, natural transmission to cattle occurred or that cattle were vaccinated against the instructions of the manufacturers or contaminated needles were used when cattle were vaccinated.

## 6. Use of SPPV and GTPX vaccines in cattle to protect against LSDV

With the introduction of LSDV into new regions which were endemic for SPPV and GPPV, the use of locally produced heterologous vaccines have been used due to political and economic reasons. The scientific rationale for using these vaccines is that these viruses are genetically related and serologically identical. However, these vaccines were used before performing efficacy studies in cattle.

A large field study in Israel compared the efficacy of RM65 SPPV vaccine with the OBP vaccine, indicating that the efficacy of the RM65 SPPV vaccine was not as effective compared to the OBP vaccine (Ben-Gera et al., 2015). An experimental study compared the efficacy of the Romania SPPV vaccine to a Neethling LSDV vaccine in cattle against LSDV challenge. This study demonstrated only partial protection with the Romania SPPV vaccine while complete protection was observed with the Neethling vaccine (Hamdi et al., 2020a,b). The extensive use of the Bakırköy SPPV vaccine in Turkey has probably contributed to some control of LSDV (Uzar et al., 2022). However, there is a clear difference with respect to the length of time required to control LSDV in Turkey, compared to Europe, which has not yet eradicated the disease. Furthermore, Russia has used the NISKHI SPPV vaccines to control LSDV in the field (Kurchenko et al., 1991), yet it has been shown to be less effective compared to the G20-LKV vaccine in protecting cattle following an experimental LSDV challenge (Zhugunissov et al., 2020).

The mechanism for the decreased efficacy of SPPV vaccines is not known, however, the immunity generated from these vaccines is not as effective as the live attenuated LSDV vaccines. The small genetic differences between antigens are unlikely to be the reason for the decreased efficacy of SPPV vaccines. It is likely that these vaccines are too attenuated and do not replicate enough in cattle to stimulate protective immune responses. Further work is required to determine why live attenuated SPPV vaccines provide partial protection against LSDV in cattle. Notwithstanding this, the NISKHI strain was capable of eradicating LSD outbreaks, caused by a classical field strain, in 2015–2016 at 100% vaccination coverage (Byadovskaya et al., 2022).

The use of GTPV vaccines to control LSDV has been demonstrated to be effective to protect cattle from experimental LSDV infection using Gorgan (Gari et al., 2015) and G20-LKV vaccines (Zhugunissov et al., 2020). Although field testing of these vaccines has not yet determined their efficacy, there are clear differences between the efficacy of live attenuated SPPV and GTPV vaccines with respect to the level of protection against LSDV in cattle. Further demonstration of the effectiveness of GTPV in the field is required to answer the question about the efficacy of live attenuated GTPV vaccines to protect against LSDV in cattle in the field.

## 7. Immunity

Lumpy skin disease induces both antibody and cellular immunity following natural or experimental infection. The level of antibody induced can depend on the severity of clinical disease with animals that have severe disease and skin lesions generally having higher levels of antibodies due to the presence of high levels of viral antigen to stimulate the B cell response. However, some cattle subclinically infected do not develop detectable levels of antibodies following infection (Tuppurainen et al., 2005; Osuagwuh et al., 2007).

It was demonstrated, using serum transfer experiments, that antibody alone could protect against capripoxvirus infections (Kitching, 1986). Unfortunately, these studies were not done using capripoxvirus specific antibodies in cattle. The role of antibody responses in protection against LSDV is further demonstrated by inactivated LSDV vaccines being able to induce antibody responses and protection following experimental infection (Wolff et al., 2020; Hamdi et al., 2020a,b).

The role of cellular immunity in the protection of cattle against LSDV is demonstrated by live attenuated vaccines being able to protect against LSDV in the absence of detectable antibody responses (Haegeman et al., 2021b). The importance of CD4+ and CD8+ T lymphocytes in the protection elicited following vaccination with live attenuated vaccines is not yet known since CD4+ and CD8+ depletion studies have not been performed in cattle.

The current experimental evidence suggests that immunity against LSDV is a combination of both antibody and cellular immune responses and that a weak response in either immunity can be compensated for.

## 8. Research gaps in the development and use of vaccines

The development of live attenuated LSDVs through either serial passage of gene deletion must not only demonstrate that the virus is safe and effective, but also that this virus will not spread and cause disease in a susceptible cattle population. The KSGPO-240 LSDV vaccine transmits and causes disease in susceptible cattle as illustrated by the LSDV outbreaks in the Indian subcontinent (Roche et al., 2020; Sudhakar et al., 2020; Kumar et al., 2021).

Despite the fact that live attenuated vaccines have been demonstrated as an effective tool to control LSDV, they are not ideal to use in countries free of the disease, due to these countries losing their freedom of disease status. This inhibits the prevention of LSDV spreading into new countries, since preventative vaccination is not practiced in countries at risk.

Although inactivated LSDV vaccines have been demonstrated to be effective in experimental trials (Wolff et al., 2020; Hamdi et al., 2020a,b), application of these vaccines offer little benefit since it is not DIVA, using current serology based assays. In addition, the ability of inactivated capripoxvirus vaccines to protect against LSDV, SPPV and GTPV has not yet been demonstrated under field conditions. Results from these trials will provide additional understanding of the similarities between the protective antibody responses against capripoxviruses.

To develop a lumpy skin disease virus DIVA vaccine requires both a vaccine and compliment diagnostic test. Currently, there are limited ELISAs which could theoretically be used as a DIVA assay (Milovanović et al., 2019). Different ELISAs currently available, could be used in future to develop DIVA companion tests, by generating novel vaccines (Heine et al., 1999; Dashprakash et al., 2019; Berguido et al., 2022).

The requirements for the development of an effective live attenuated DIVA vaccine are: Having validated companion diagnostic ELISAs that can differentiate infected from vaccinated animals (2 tests, one to identify vaccinated animals and one to identify vaccinated animals which have been infected); Being able to delete the antigen encoded gene from one of the validated diagnostic ELISAs in a live attenuated vaccine. This requires the specific gene to be non-essential for viral replication. Following successful generation of the gene deleted live attenuated vaccine, this vaccine must be able to still induce protective immunity. If the deleted antigen is required to induce a protective immune response then the vaccine will not be effective. Finally, vaccinated animals following exposure to the virus are required to induce an antibody response to the antigen used in the companion diagnostic test.

An alternative approach to develop a DIVA vaccine is to identify the antigen(s) of LSDV that elicit a protective immune response, which are currently unknown. However, the complicating factor with LSDV is the numerous antigens present which makes identification difficult compared to many other viruses. In addition, it is not known if a single or more antigens are able to elicit a protective response against the virus. Once these antigen(s) have been identified there are many different vaccine platforms including subunit, mRNA and adenoviral vectors (Riel and de Wit, 2020). which could be used to develop an alternative effective vaccine for LSDV.

Another critical point for consideration is the consequences of the use of live attenuated vaccines (Hanley, 2011; Bull, 2015). Since they are replication-competent there is a risk for a single cell to be coinfected by two different parental genotypes. The recombination in general for capripoxviruses was only a hypothesis before 2017 (Gershon et al., 1989; Krotova et al., 2022a). The recent insights into the LSDV molecular epidemiology exhibited the involvement of Neethling and KSGP vaccine strains in the emergence of virulent vaccine-like strains with novel features reviewed here (Sprygin et al., 2018a). It is now evident that recombination in poxviruses occurs at a greater scale than thought and by mechanisms that are unique to poxviruses only (Evans, 2022).

Importantly, recombination is troubling not only for its contribution to virulence, but also because of its potential to generate new species of poxviruses. For example, Malignant rabbit virus (MRV) is the result of recombination between myxoma virus and Shope fibroma virus (Sprygin et al., 2022). It is hypothesized that capripoxviruses diverged from a common ancestor through both genetic drift and recombination at different rates (Gershon et al., 1989; van Schalkwyk et al., 2022). Molecular clocks calculated for capripoxviruses for the first time in history showed that LSDV as a species appeared about 500 years ago (van Schalkwyk et al., 2022).

## 9. Global molecular epidemiology

Understanding molecular epidemiology is a necessity for implementation of control strategies. Complete genome sequencing of an isolate provides in depth analysis of phylogenomic relationships, evolutionary changes and molecular epidemiology related to an outbreak. Genetic characterization and phylogenetic analysis during outbreaks of LSDV aids in understanding several aspects, for example, disease hotspot areas, level of transboundary circulation, origin of LSDV and detection of any new variants (Ochwo et al., 2020).

Since LSDV was first described in 1929 in Zambia, the genome of the virus was stable, illustrated by genetic analysis of field isolates from Africa displaying only minor differences. Furthermore, a comparison of field isolates from the Middle East in 2012 and Europe in 2015 to African isolates also demonstrated the genetic stability of LSDV (Stram et al., 2008; Agianniotaki et al., 2017a).

The genome of LSDV is about 150 kbp in length composed of a central coding region containing about 156 ORFs and sandwiched by identical inverted terminal repeats about 2.4 kbp in length each. Complete genome comparisons between the genome of LSDV to chordopoxviruses of other genera, indicated 146 genes conserved over the species. At the same time comparing LSDV central coding region to other known mammalian poxviruses (*Suipoxvirus, Yatapoxvirus, and Leporipoxvirus*) indicated colinearity and amino acid conservation of sequence identity up to 65%, yet this conservation decreases to 45% or even 0% when comparing the inverted terminal repeats (Tulman et al., 2001). In 2017, a novel recombinant was isolated from an active outbreak in Saratov, Russia. Genomic characterization of the virus indicated that it had a backbone of a Neethling/LW1959 vaccine strain, with 27 major recombination events involving a KSGPO-like vaccine integrated throughout (Sprygin et al., 2018a). A virus with high sequence identity to Saratov/2017 was isolated in Saratov in late 2019, indicating the ability of the novel recombinant LSDV to overwinter in the freezing winter conditions of Russia (Shumilova et al., 2022a). A second novel recombinant LSDV strain was isolated and characterized in March 2018 in Udmurtia, Russia, during frozen winter conditions (Sprygin et al., 2020). In contrast to the first recombinant, Saratov/2017, the second LSDV (Udmurtia/2018) had a genomic backbone identical to KSGPO-like strains with portions of Neethling/LW1959 contained within due to unique recombination events between the two strains (Sprygin et al., 2018a). These two novel recombinants were genetically unique and appeared to be restricted to localized outbreaks within a specific region (Shumilova et al., 2022b).

A third novel recombinant was identified in Tuymen, Russia in 2019 with a genomic backbone identical to the Neethling vaccine with patterns of KSGPO-like vaccine contained within (Krotova et al., 2022b). This was soon followed by the description of the fourth novel recombinant strain in China from 2019 (Lu et al., 2020). The virus spread rapidly across China to neighboring countries including Vietnam, Cambodia, Laos, Mongolia, Russia and Thailand (Seerintra et al., 2021; Tran et al., 2021; Sprygin et al., 2022; Suwankitwat et al., 2022; Krotova et al., 2022a). This is currently the dominant lineage spreading in southeast Asia, including Malaysia, Singapore and Indonesia.

### 9.1. Molecular analysis based on full-genome sequencing

**Figure 1:**
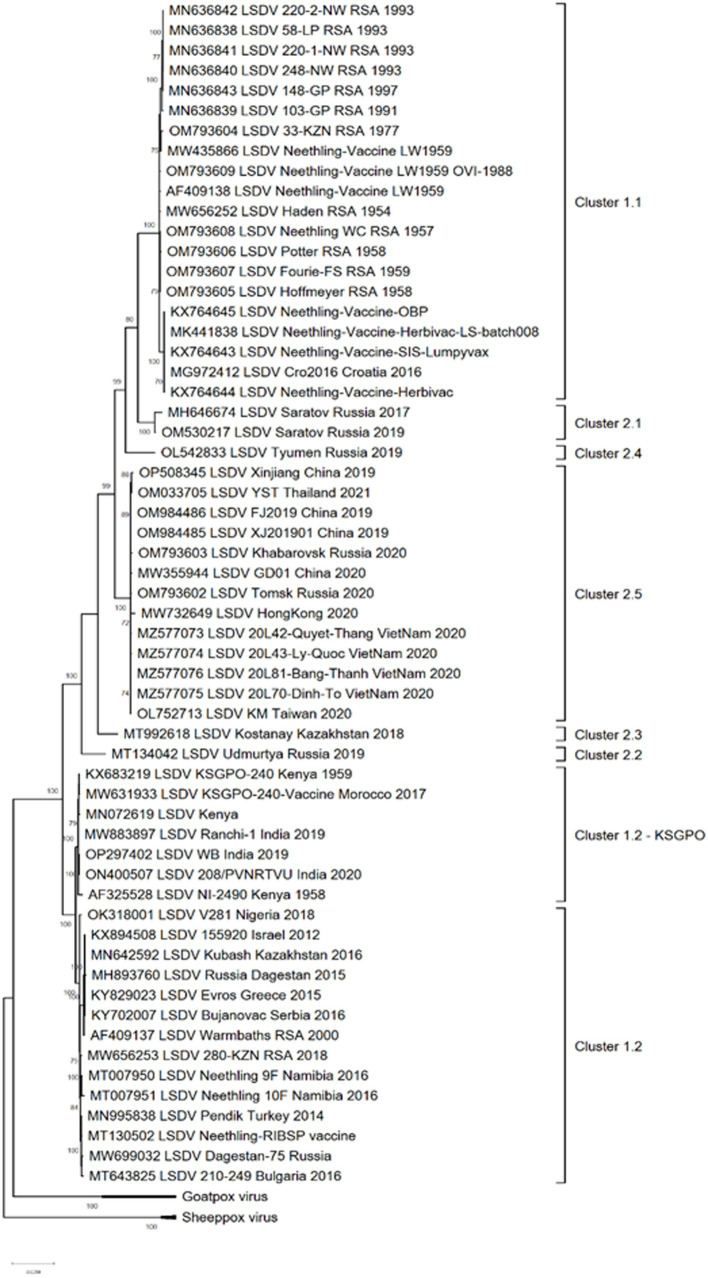
Maximum likelihood phylogenetic analysis based on full genome sequences of LSDV isolates obtained from Genbank and representing all the groups and clusters of the virus.

Complete genome sequencing of LSDVs obtained from outbreaks in new geographic regions or representing isolates with differences in pathogenicity or transmission phenotype, is required to investigate virus evolution and gene selection. Genetic changes can be further characterized to confirm their effect on the phenotype. Currently, 59 complete genome sequences of LSDV isolates are available in Genbank, representing vaccine strains and viruses isolated from several countries where the virus circulates (Figure 1). These sequences represent isolates from Africa, the Middle East, Europe, Russia and Asia.

The phylogenetic analysis of these isolates divided them into three major groups; clusters 1.1, 1.2 and the new cluster 2 consisting of novel recombinants. The latter cluster is subdivided into five separate clusters, designated 2.1 to 2.5, with the majority of these sub-clusters consisting of sequences from one or two isolates (Figure 1).

As demonstrated in Figure 1, clusters 1.1 and 1.2 consist of isolates from different regions of the world as well as a large temporal distribution. The earliest isolate is from South Africa in 1954, whilst the newest from India in 2020 (Kumar et al., 2021; van Schalkwyk et al., 2022). In contrast, the five sub lineages of novel recombinant strains constituting cluster 2 were detected from 2017 and only in Russia, Kazakhstan and Asian countries (Sprygin et al., 2018a, 2020; Krotova et al., 2022a,b,c).

**Figure 2:**
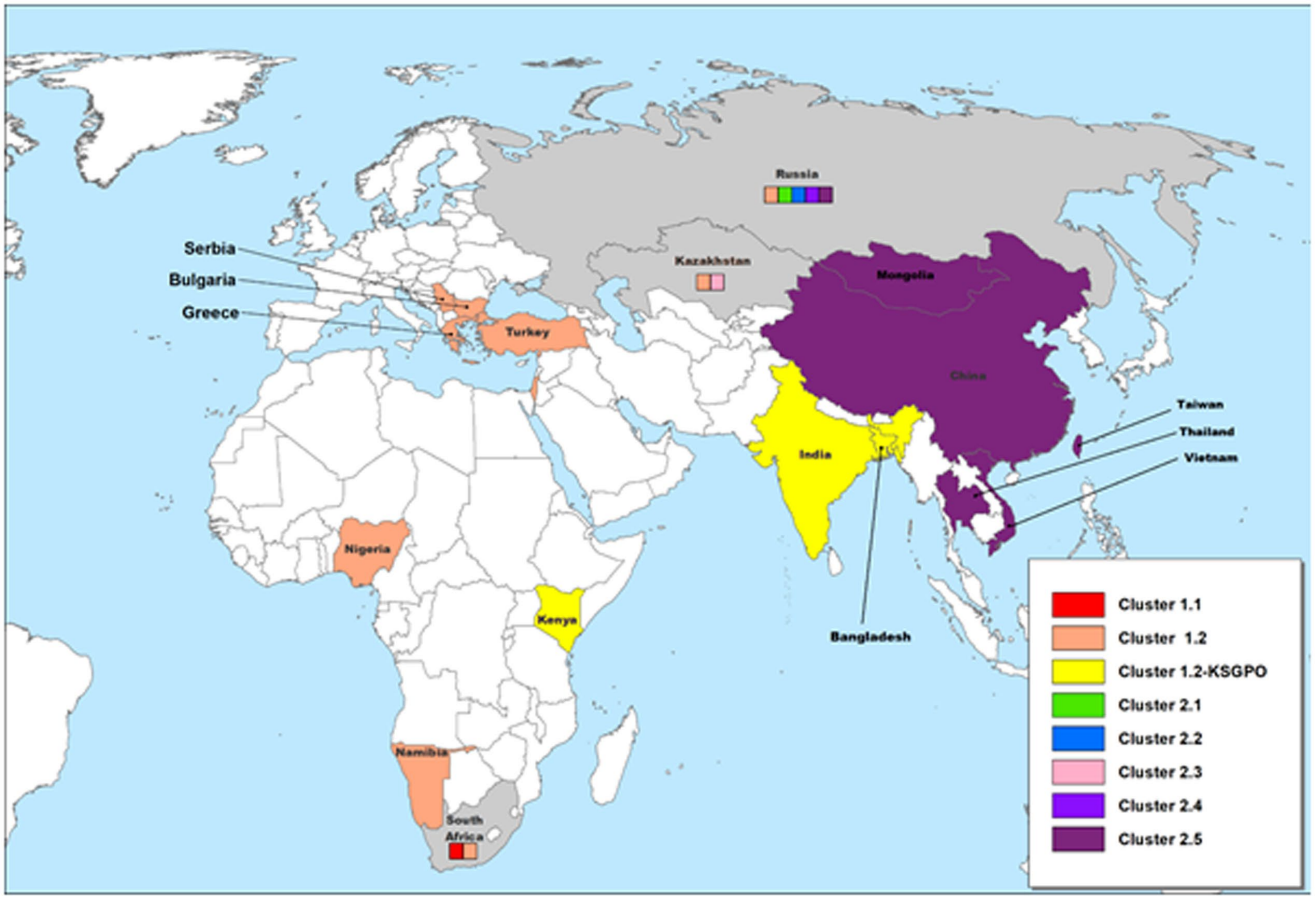
Geographic distribution of different LSDV clusters based on the full genome sequences of isolates from these regions. Countries where more than one phylogenetic cluster were identified are indicated in gray, whilst the different clusters present in these countries are included in small colored squares (each colored square represents a different cluster). This map represents outbreaks from 1954 to 2022.

The geographical distribution of isolates whose complete genomes have been elucidated are indicated in Figure 2.

**Figure 3:**
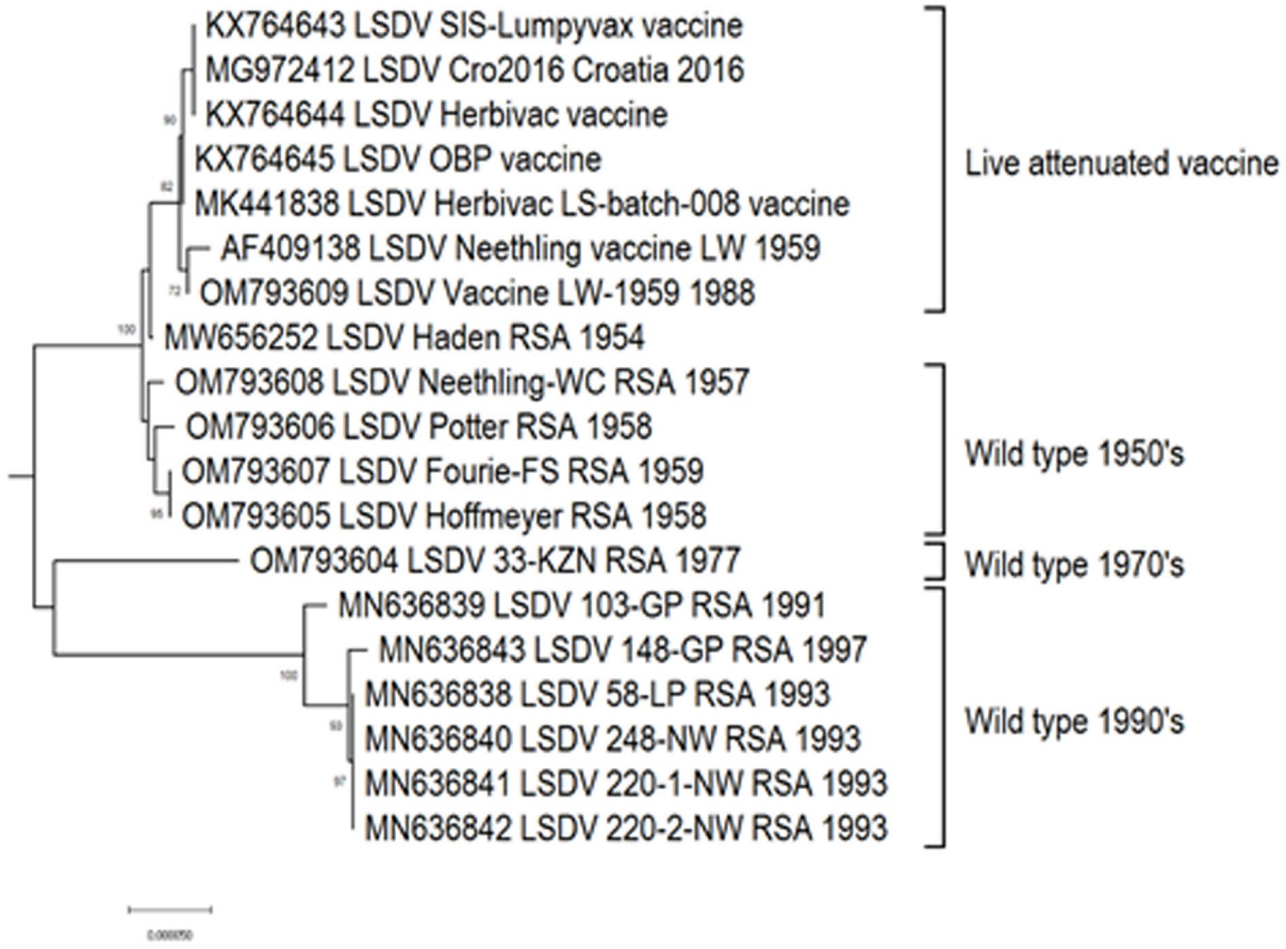
Maximum likelihood phylogenetic tree representing the complete genomes of South African isolates and Neethling-LW1959-based vaccines belonging to Cluster 1.1.

Based on complete genome sequences, it has been calculated that clusters 1.1 and 1.2 shared a common ancestor around 550 years ago (van Schalkwyk et al., 2022). The first characterized isolates representing both these clusters were circulating, respectively, in South Africa (1.1) and Kenya (1.2) in the 1950’s (Tulman et al., 2001; van Schalkwyk et al., 2022). Cluster 1.1 is represented by the LSDV type strain Neethling, isolated in 1957 and attenuated to a commercial vaccine in 1959 (Alexander et al., 1957; van Rooyen et al., 1959). Seven nucleotide differences were detected between the virulent Neethling/1957 and vaccine/1959 strains (van Schalkwyk et al., 2020). The currently available commercial vaccines based on this isolate, are grouped into the same cluster with minimal nucleotide differences between them, indicating stable maintenance of the vaccine stock (Kara et al., 2003; Mathijs et al., 2016; Douglass et al., 2019). Additionally, Cluster 1.1 contains virulent field isolates from South Africa isolated in the 1970’s and 1990’s, with 69 nucleotide differences within this cluster, possibly due to genetic drift (Figure 3; van Schalkwyk et al., 2020, 2022).

**Figure 4:**
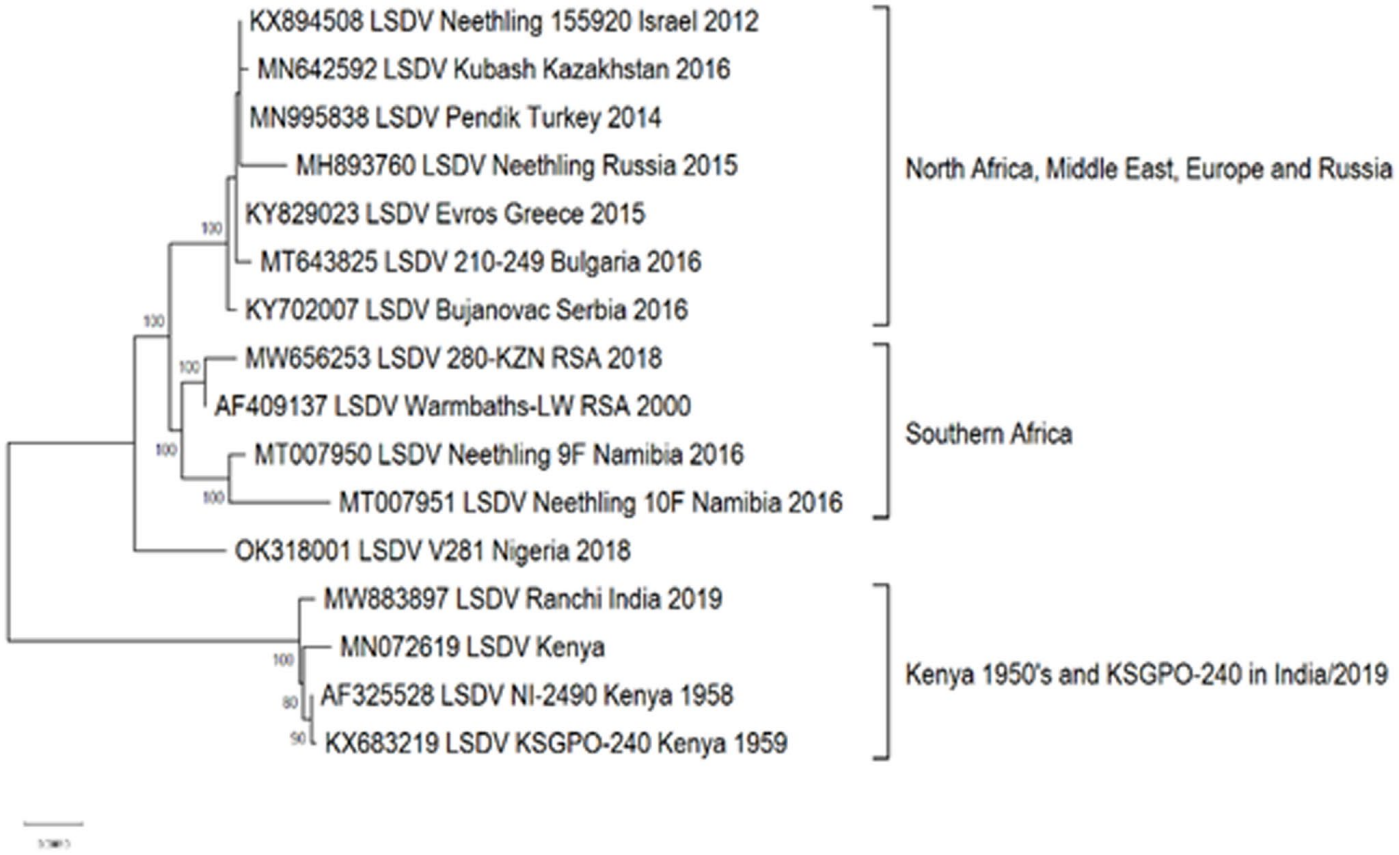
Maximum likelihood phylogenetic tree representing the complete genomes of isolates belonging to Cluster 1.2.

Approximately, 2,200 nucleotide differences were described between isolates belonging to clusters 1.1 and 1.2 (Kara et al., 2003). In contrast to the limited geographical distribution of Cluster 1.1, currently consisting of isolates only from South Africa, Cluster 1.2 has samples from Africa, the Middle East, Europe and Asia (Figure 4). The sequences contained in this cluster could be subdivided into three sub-lineages, based on ~260 nucleotide differences between them (Figure 4). The basal lineage consists of the original NI-2490 and KSGPO-240 from Kenya in 1958 (Capstick, 1959), as well as the KSGPO-240 vaccine isolates from India, Nepal and Bangladesh in 2019–2020 (Figure 4). The second cluster contains isolates from sub-Saharan Africa, including sequences from Namibia and South Africa, with the latter only since 2000 (Di Felice et al., 2020; van Schalkwyk et al., 2022). In contrast, the third sub-cluster has a short temporal, but vast spatial distribution. Isolates from the Middle East, Europe and Asia represent outbreaks from 2010 to 2016 (Agianniotaki et al., 2017a,b; Sprygin et al., 2019b).

As illustrated in Figure 2, isolates from Cluster 1.1 were restricted to a specific region, whilst isolates belonging to Cluster 1.2 were isolated in several countries starting from South Africa in Africa, to Kazakhstan and the Russian Federation in Asia, Israel in the Middle East and the EU countries (Greece, Serbia and Bulgaria). These isolates have been circulating for more than 70 years with a high level of genome stability. In contrast, isolates belonging to cluster 2 are already distributed to 5 different clusters due to the high level of intergenic recombination occurring across the genomes of these viruses, despite the first description only in 2017 (Sprygin et al., 2018a, 2020; van Schalkwyk et al., 2022).

**Figure 5:**
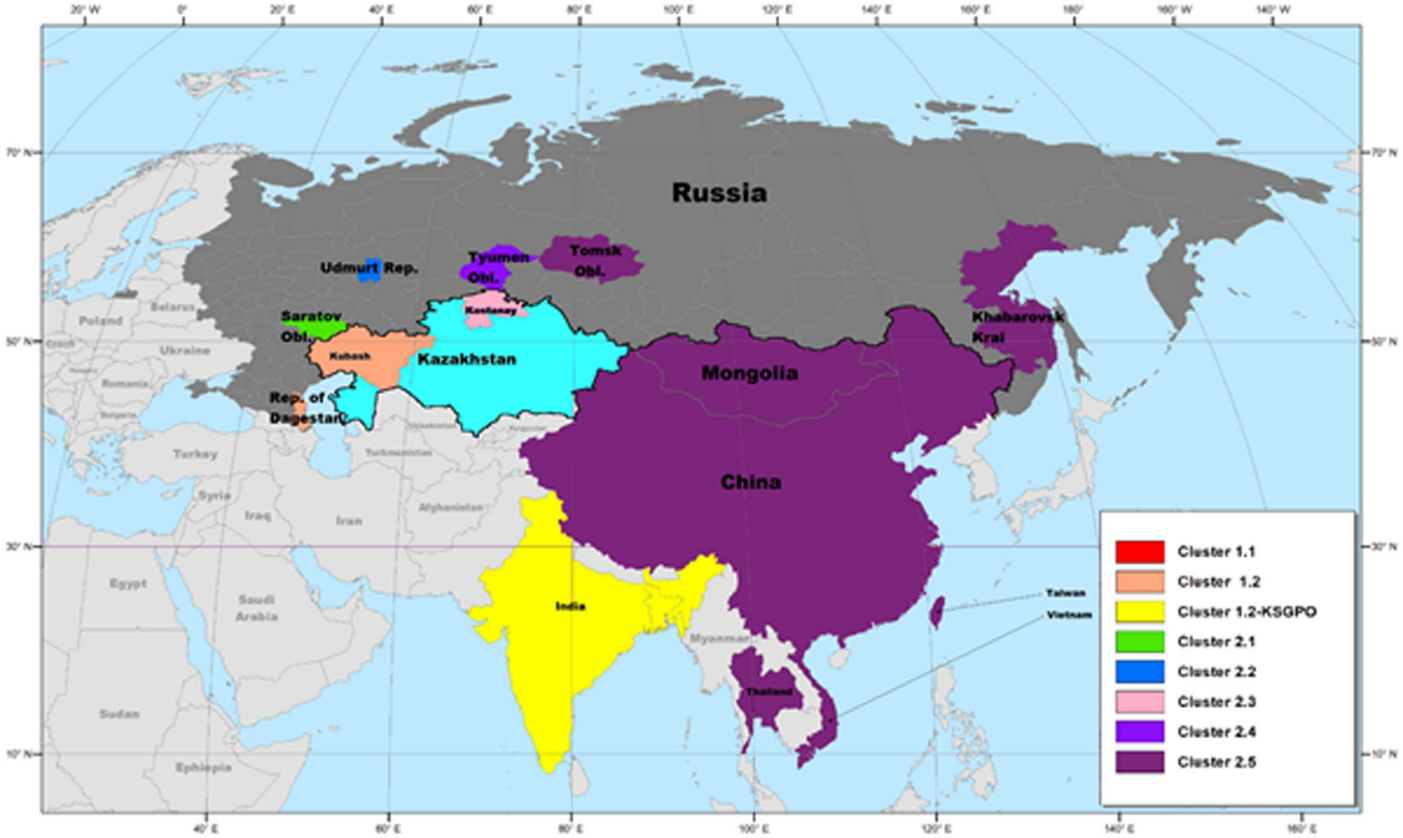
Geographic distribution of LSDV strains circulating in Asia. The dominant lineage in South-East Asia is the recombinant Cluster 2.5, whilst the vaccine KSGPO-240 is causing outbreaks in the Indian subcontinent. This map represents outbreaks from 2015 to 2022.

**Figure 6:**
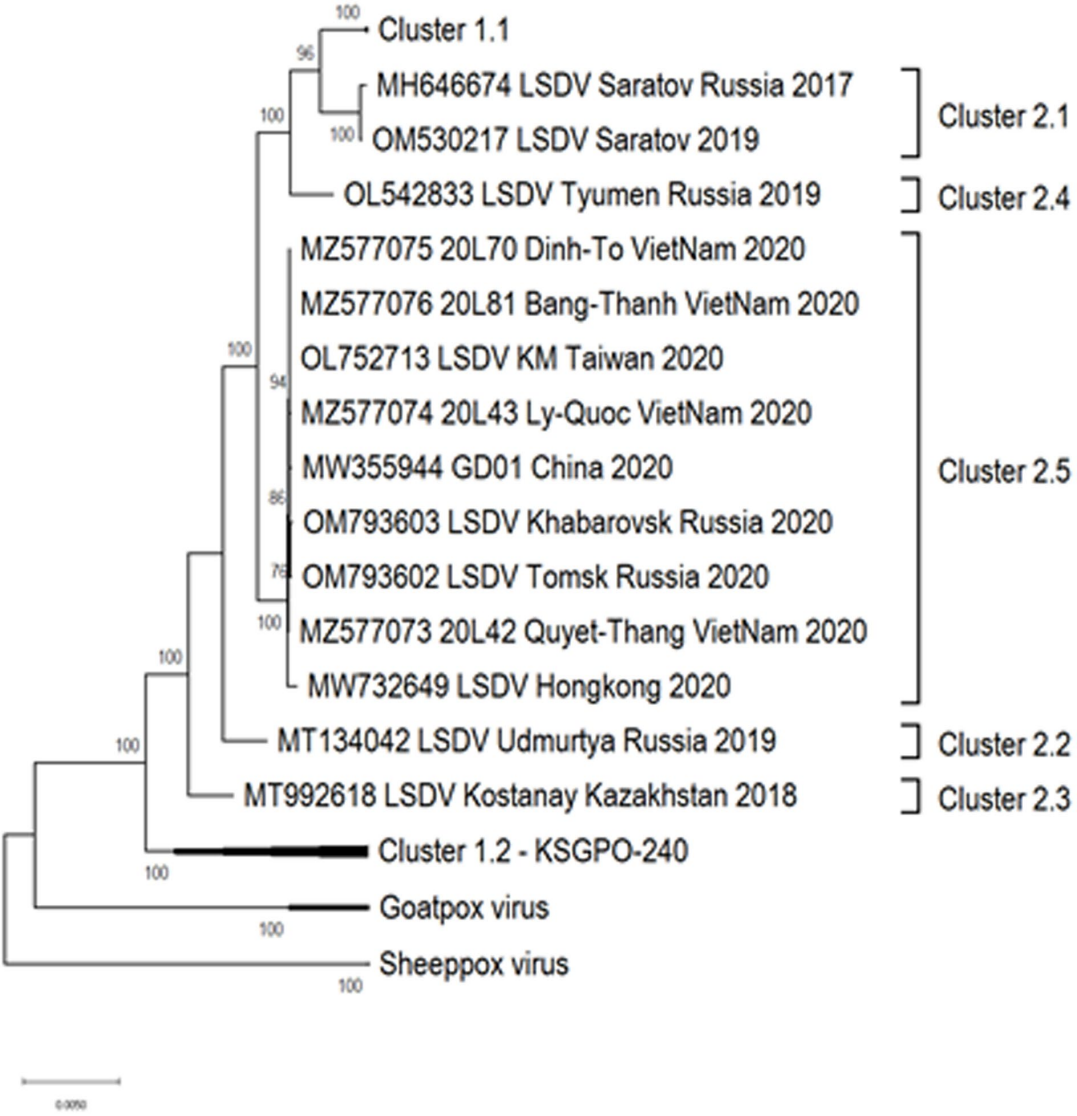
Maximum likelihood phylogenetic tree representing the complete genome sequences of isolates belonging to Cluster 2 in relation to their parental strains in Cluster 1.1 and Cluster 1.2.

**Figure 7:**
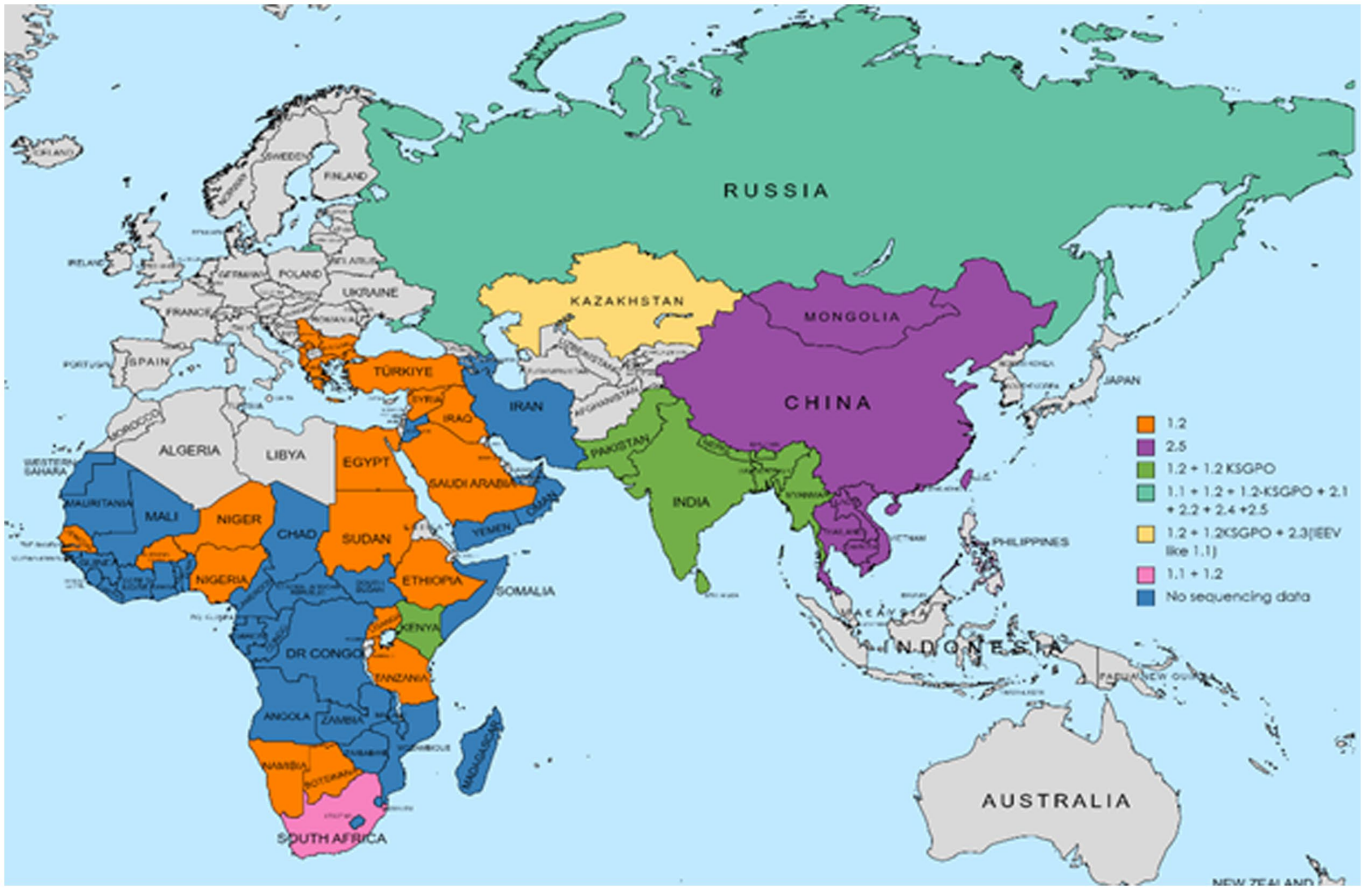
World wide distribution of LSDV based on available information of sequenced genome markers. This map represents outbreaks from 1929 to 2022.

As previously mentioned, the isolate from Saratov in Russia in 2017 was the first novel recombinant strain to be described and formed with a second isolate from the same region in 2019 in cluster 2.1 (indicated with green in Figure 5). These viruses had 60% of the SNPs identical to the Neethling-LW1959 vaccine and the remaining 40% were identical to KSGPO-240, thus sharing a close common ancestor to Cluster 1.1 (Figure 6; Sprygin et al., 2018a; Krotova et al., 2022a; Shumilova et al., 2022b). Cluster 2.2 consists of the second recombinant strain identified in Udmurtiya in Russia, in 2019, indicated with light blue in Figure 5 (Sprygin et al., 2020). The isolate LSDV/Russia/Udmurtiya/2019 was genetically different from Saratov/2017, except for the shared parental strains Neethling-LW1959 vaccine and KSGPO-240. This isolate shared 55% of the SNPs with KSGPO-240 and only 45% with Neethling-LW1959 vaccine resulting in a closer phylogenetic association to Cluster 1.2 (Figure 6; Krotova et al., 2022a). The complete genome sequence of a third unique novel recombinant strain Kostanay/Kazakhstan/2018 was submitted to GenBank, but no additional information pertaining to this isolate was published (Figure 5, dark blue). Despite also sharing 55% of the SNPs with KSGPO-240 and 45% with Neethling-LW1959 vaccine, the complete genomes of Kostanay/Kazakhstan/2018 and LSDV/Russia/Udmurtiya/2019 are not identical, resulting in Kostanay/Kazakhstan/2018 forming into Cluster 2.3 (Figure 6; Krotova et al., 2022a). Cluster 2.4 contains yet another novel recombinant from Russia in 2019, LSDV/Russia/Tyumen/2019 (Figure 5, light purple). It shares 38% of the SNPs with KSGPO-240 and 62% with Neethling-LW-1959 vaccine, resulting in a closer phylogenetic relationship to Cluster 1.1 (Figure 6; Krotova et al., 2022a,b). The last lineage to be identified in 2019, Cluster 2.5, was first described in an outbreak in the Xinjiang region of China in 2019 (Lu et al., 2020). Viruses belonging to this lineage, indicated in dark purple in Figure 5, have spread across China south to Vietnam, Taiwan, Thailand, Cambodia, Malaysia and Indonesia as well as north to Mongolia and Russia in 2020 (Figures 5, 7). The dominant lineage has 48% of the SNPs identical to KSGPO-240 and 52% identical to Neethling-LW-1959 vaccine (Figure 6; Krotova et al., 2022a). It is interesting to note, that during the same time novel recombinant Cluster 2.5 were spreading across South-East Asia, one of its parental strains, the vaccine strain KSGPO-240, were introduced and spread across Bangladesh, India, Myanmar, Nepal, Bhutan, Pakistan and Sri-Lanka. As yet, no subsequent recombination between recombinant cluster 2.5 and parent vaccine KSGPO-240 strains has been reported (Figure 7).

### 9.2. Molecular analysis based on sequencing of specific genome markers

Complete genome sequencing of an isolate provides in depth analysis of phylogenomic relationships, evolutionary changes and molecular epidemiology related to an outbreak. Since complete genome sequencing requires specialized infrastructure and equipment it is still expensive, laborious and not attainable by many laboratories. The complete genome of LSDV exceeds 150 kbp in length, rendering full genome sequencing of each isolate impractical, yet an affordable and easy method to characterize a large representative of an outbreak is to sequence several genome loci. In the case of LSDV this is based on sequencing four genes; GPCR, RPO30, P32 and EEV (Lamien et al., 2011; Ochwo et al., 2020; Sprygin et al., 2020; Sudhakar et al., 2020; Badhy et al., 2021).

#### 9.2.1. P32

The WOAH-approved molecular test to identify capripoxviruses was developed by Ireland and Binepal in 1998. It is based on the 192 bp region of the LSDV ORF LW074, encoding the P32 intracellular mature virus (IMV) envelope protein. Due to the small size of this amplicon and the limited SNPs contained within, it only divides LSDV into four clusters, 1.1, 1.2, 2.1, and 2.4 (Table 1). Based on the sequences of P32, clusters 2.2 and 2.3 (Udmurtiya/Russia/2019 and Kostanay/Kazakhstan/2018) grouped with Cluster 1.1, whilst Cluster 2.5 (represented by GD1/China/2019) grouped with cluster 1.2.

**Table 1:** Capability of genome markers to detect recombinant LSDV strains.

	Genome marker			
Recombinant Cluster	P32	GPCR	RPO30	EEV
2.1	+	−	−	−
2.2	−	+	+	+
2.3	−	−	+	−
2.4	+	+	−	−
2.5	−	+	−	−
2.6 (New possible group)	−	+	+	+

#### 9.2.2. GPCR

The G-protein-coupled chemokine receptor (GPCR) gene, located in ORF LW011 was identified as a gene target which could be used for differentiating between the three capripoxviruses (Le Goff et al., 2009). Since it is a large region and contains various polymorphisms, in addition to identifying the capripoxvirus, it can be used to cluster viruses into individual sub-lineages. Currently, this marker clusters LSDV isolates into seven clusters, 1.1, 1.2, 2.2, 2.3, 2.4, 2.5 and a possible new recombinant Group 2.6 (Table 1). Unfortunately, it does not discriminate between sequences in Cluster 1.2 and 1.2-KSGO and additionally, Saratov/Russia/2017 (2.1) is clustered with 1.1 (Sprygin et al., 2018a).

#### 9.2.3. RPO30

The genome marker RPO30 is based on ORF LW035 that encodes the 30 kDa subunit of the RNA polymerase. Originally designed to differentiate between the three capripoxviruses, it was soon implemented to contribute to the molecular epidemiological characterization of LSD outbreaks (Lamien et al., 2011). The genetic variability within each of the capripoxviruses is generally low, thus any nucleotide sequence variation is highly indicative of a true difference between isolates, i.e., vaccine strains versus field isolates, or in the case of LSDV the difference between Cluster 1.1 and 1.2 (Molini et al., 2018). Amplification, sequencing and phylogenetic analysis of a 486 bp region of the ORF, enables the clustering of all the known LSDV isolates into six clusters, i.e., Clusters 1.1, 1.2, 1.2 KSGP, 2.1, 2.4 and 2.5 (Table 1). This implies that two of the previously described novel recombinant clusters (2.2 and 2.3) were not identified as individual clusters (Sprygin et al., 2020). The sequences of Udmurtiya/Russia/2019 (2.2) clusters with 1.2-KSGO, whilst Kostanay/Kazakhstan/2018 clusters with Saratov/Russia/2017 in Cluster 2.1.

#### 9.2.4. EEV

The extracellular enveloped (EEV) glycoprotein, encoded by LSDV ORF LW126, is preferentially used as a marker to differentiate between vaccine (Neethling-LW1959) and field isolates belonging to Cluster 1.2 (Menasherow et al., 2014). This DIVA strategy is based on the amplification of a 1,051 bp fragment of the ORF, subsequent sequencing and comparison to the vaccine strains. The EEV glycoprotein gene sequence has a 27-nucleotide insertion in the genome of field isolates (Cluster 1.2) in comparison to vaccines in Cluster 1.1. These 27 bps are present in all isolates of Cluster 1.2, including the KSGP-0240 derived vaccines and historical NI2490 (1958) and LSDV Kenya (1950) sequences from Kenya. As a marker for epidemiological clustering of isolates, this marker has the ability to differentiate isolates into five clusters, i.e., 1.1, 1.2, 1.2-KSGP and two novel recombinant clusters 2.2 and 2.5. The remainder of the novel recombinant viruses belong to Cluster 1.1.

The majority of the previously described markers were designed to either identify the capripoxvirus species or to differentiate vaccine and field viruses, however, they all discriminate between isolates belonging to clusters 1.1 and 1.2. Comparison of the genomes of recent virulent field viruses belonging to Cluster 1.1 and novel recombinant viruses, demonstrated these markers could not accurately identifying recombinant viruses (Sprygin et al., 2018a, 2020; Ma et al., 2022; van Schalkwyk et al., 2022; Krotova et al., 2022a,b). In addition to the recombinant strains identified in Russia, Kazakhstan and South-East Asia, a description of a single cattle vaccinated in Kenya (Embu/B338/2011) with mixed features, has been described (Chibssa et al., 2021). Based on sequence analysis of the partial PRO-30, GPCR, EEV and B22R genes, the field virus LSDV Embu/B338/2011 from displayed features of both LSDV Neethling vaccine and field isolates using these markers, although no full genome was provided (Chibssa et al., 2021). A summary of the ability of each marker to identify the novel recombinant strains, are provided in Table 1.

As demonstrated in Table 1, not a single genome marker harbors enough information to differentiate between the recombinant isolates, yet used in combination the markers could identify the currently recognized recombinant strains, but might misidentify novel strains.

For this reason Krotova et al. (2023), suggested using a single marker capable of differentiation and identification between all the different recombinant clusters. The authors identified a region in open reading frame (ORF) LW134, that is 705 bp in size. Where 13 informative single nucleotide polymorphisms (SNPs) were capable of segregating the novel recombinant vaccine-like strains RVLSs accurately into previously designated clusters, based on complete genomes sequences. This assay is based on a single PCR reaction followed by DNA sequencing to identify previously described recombinant strains and cluster them into pre-identified groups.

## 10. Similarities and differences between classical and recombinant vaccine-like LSDV

The impact of an LSDV outbreak, in a new region, on the cattle industry is severe regardless of the strain of the virus. The clinical signs and severity of the disease are similar between classical LSDV, KSGPO and recombinant LSDV strains. In outbreaks these viruses display variability in morbidity, mortality and severity of clinical disease. The patterns of virus replication and secretion between experimental infection of classical LSDV (Babiuk et al., 2008b) and recombinant LSDV observed in the field are similar (Li et al., 2022).

Differences between the recombinant viruses and classical LSDV and KSGPO are the improved transmission by contact with recombinant viruses. The molecular mechanism for this is not understood. All three lineages (Figure 1) have demonstrated the ability to spread into new geographic regions as these viruses can be spread over distance by vectors.

To date, there is no evidence that recombinant or KSGPO LSDV isolates can evade control by safe and effective live attenuated vaccines.

## 11. DIVA diagnostics

Rapid and reliable diagnostic tools have always been instrumental for disease control and eradication. The initial PCR assays on LSDV were conventional PCR approaches targeting capripoxvirus genus specific genes (Ireland and Binepal, 1998), followed by real-time PCR to detect capripoxviruses (Bowden et al., 2008). The geographic expansion of LSDV beyond Africa into the Middle East and Europe and the use of live attenuated LSDV vaccines required a diagnostic to be able to differentiate LSDV between field isolates and the live attenuated vaccines used. Different approaches were used that identified genetic differences between the circulating LSDVs in the field, predominantly belonging to Cluster 1.2, and the commercially available live attenuated vaccines based on Neethling-LW1959 (Vidanović et al., 2016; Agianniotaki et al., 2017b; Erster et al., 2019; Sprygin et al., 2019a,b,c). These DIVA diagnostics were fit-for-purpose prior to the emergence of recombinant LSDVs, even though these diagnostics did not target specific LSDV genes critical for the virulence of field isolates.

With the emergence of recombinant vaccine-like LSDVs, the DIVA diagnostics became problematic because the genomic rearrangements in LSDV genomes due to recombination caused a breakdown in the performance of the all existing assays and kits (Byadovskaya et al., 2021). The currently established lineages in South East Asia cluster 2.5 is detected as vaccine by two assays: the GPCR gene based real time PCR (Agianniotaki et al., 2017a,b) and real-time PCR assay targeting the vaccine LSDV strain (Sprygin et al., 2018b), whereas the KSGP strains from India and Bangladesh are detected positive by the GPCR assay developed by Agianniotaki and negative by the vaccine assay developed by Sprygin (Agianniotaki et al., 2017a,b; Sprygin et al., 2019c).

Interestingly, virulent strains like Haden/RSA/1956 and the prototype strain Neethling/RSA/1957, circulating in South Africa before 2000, belonged to cluster 1.1. If tested using the aforementioned DIVA assay, they would test positive using both the vaccine Agianniotaki GPCR assay and the vaccine Sprygin 008 (Agianniotaki et al., 2017a,b; Sprygin et al., 2019c).

The current understanding of molecular epidemiology objectively necessitates a need for further efforts in the search for proper genetic targets related to virulence and transmission to differentiate the current lineages using PCR.

Based on analysis of full-genome sequences of the RVLS of LSDV researchers are developing new RT-PCR kits that can differentiate between field isolates, vaccine strains and RVLS. Krotova et al. (2023) identified a 705-bp region in open reading frame (ORF) LW134 that can be used for this purpose. Based on a single run of nucleotide sequencing and phylogenetic analysis, the region with 13 informative single nucleotide polymorphisms (SNPs) was capable of accurately segregating the novel RVLSs into the same five clusters previously confirmed by whole-genome sequencing (Krotova et al., 2023). Haegeman et al. (2023a,b) of a validated RT-PCR allowing to differentiate wild-type LSDV strains, including the Asian recombinant strains, from Neethling-based vaccine strains (Haegeman et al., 2023a,b). This work was directly followed by a research validating another RT-PCR allowing to targeting open reading frame LW032, capable of specifically detecting KSGP-related isolates and recombinant LSDV strains containing the KSGP backbone (Sprygin et al., 2023).

## 12. Conclusion

LSDV is continuing to spread into new regions and still poses multiple challenges both to farmers and policy makers, complicating its control and eradication in livestock and wildlife. The ability of LSDV to spread into new regions has historically been underestimated (Roche et al., 2020). The current spread of LSDV into most of Asia continues to threaten Southeast Asia and Australia. In addition, since LSDV can easily transmit in naive cattle, an outbreak in the Western hemisphere would likely spread uncontrollably in Central or South America. For these reasons LSDV continues to be an emerging global threat to cattle (Babiuk et al., 2008a). South African researchers first established that LSDV could be transmitted *via* contact, shared water and vectors, with recent investigations only echoing the original observations at a more sophisticated laboratory level. Current observations from around the globe confirm these findings and give further impetus to studying the overwintering capacity of LSDV not only under tropical winters but also freezing winters with snow cover.

The genesis of recombinant LSDVs occurring as a result of multiple homologous recombination events between the two widely used vaccine strains Neethling and KSGP gave rise to a plethora of unique virus variants, followed by positive selection of the fittest currently established in South Easten Asian countries and represented by the single lineage cluster 2.5.

Of note, the cluster 2.5 viruses are the lineage spreading across the region, with cluster 1.2 KSGP viruses radiating out from India possibly due to the use of the KSGP based vaccine (KS-1) against sheep pox with an escape and spillover into susceptible cattle populations in India and spreading to neighboring countries. The future spread of these viruses between regions is not known. It is possible that either both viruses cluster 2.5 and cluster 1.2 continue their spread into neighboring regions or that one cluster becomes the dominant virus.

The lack of fit-for-purpose DIVA assays to properly distinguish virulent viruses and live attenuated vaccines remains a research gap. The previously used approaches for DIVA assays using a small range of genomic sequences of only classical and vaccine strains, were not effective for use with the emergence of the recombinant LSDVs. New approaches using genes required for virulence are needed to discriminate between live attenuated Neethling vaccines and virulent LSDV viruses.

## Author contributions

AM: Investigation, Methodology, Software, Writing – original draft, Writing – review & editing. AV: Conceptualization, Validation, Visualization, Writing – original draft, Writing – review & editing. SB: Writing – original draft, Writing – review & editing. EV: Writing – original draft, Writing – review & editing. DW: Writing – original draft. AS: Conceptualization, Data curation, Funding acquisition, Writing – original draft, Writing – review & editing.
